# *Salmonella enterica* Serovar Typhimurium Slows Down to Dodge Antibiotics

**DOI:** 10.1371/journal.pbio.1001794

**Published:** 2014-02-18

**Authors:** Caitlin Sedwick

**Affiliations:** Freelance Science Writer, San Diego, California, United States of America

Today, widespread antibiotic resistance means bacterial infections pose a serious threat to human health. Of course, not all infections are lethal; for example, many people infected by the pathogenic *Salmonella enterica* serovar Typhimurium (*S.* Tm.) experience only a bout of diarrhea. However, some people develop “complicated” *S.* Tm. infections, in which the bacteria spread systemically and provoke life-threatening disease. These infections are aggressively treated with potent antibiotics such as the fluoroquinolone ciprofloxacin, but sometimes even a full course of ciprofloxacin treatment (5–7 days' duration) fails to fully eradicate the bacteria from the body. Therefore, upon cessation of antibiotic treatment, patients experience relapses of the infection. It's unknown how or why this occurs, so Patrick Kaiser, Roland Regoes, Wolf-Dietrich Hardt, and colleagues studied the mechanisms of complicated *S.* Tm. relapses in their paper published this month in *PLOS Biology*.

**Figure:**
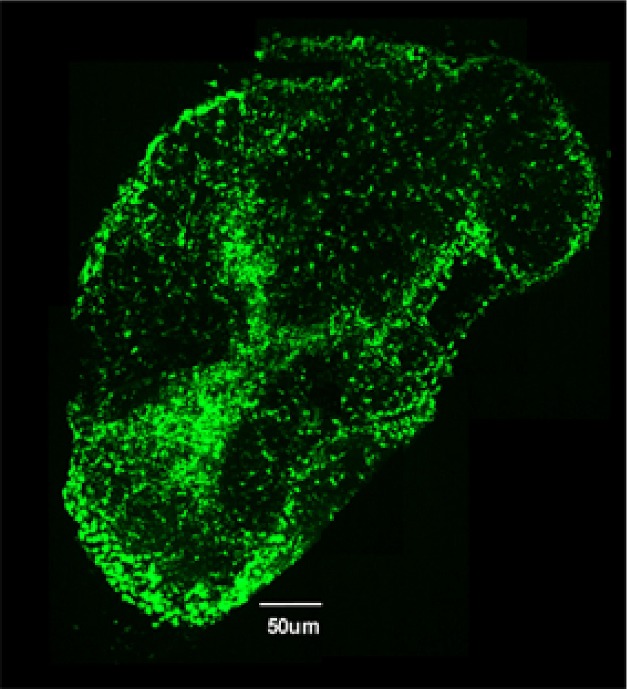
Image of a cecum draining lymph node (cLN) from a mouse infected with *S.* Tm. and treated for two days with ciprofloxacin. Green fluorescence marks the “classical” dendritic cells present in the cLN.

To explore *S.* Tm.'s response to antibiotic treatment, Kaiser and colleagues studied the course of infection in C57BL/6 mice. These mice develop diarrhea and systemic infections resembling complicated *S.* Tm. cases in humans. And, just as in humans, the animals' symptoms improve with ciprofloxacin treatment, but relapse upon withdrawal of the antibiotic.

The authors' experiments showed that high doses of ciprofloxacin treatment quickly (within three hours) eradicated bacteria from the animals' gut lumen. The antibiotic also quickly reduced bacterial levels within a pouchlike region of the gut called the cecum, and within the lymph node associated with that tissue (the cecum draining lymph node, or cLN). But within two hours of treatment initiation, the rate of bacterial killing in cecal tissue and the cLN was observed to drop precipitously. As a result, viable *S.* Tm. cells were still present after ten days of treatment, with the highest bacterial concentrations in the cLN, but with bacteria also detectable in other tissues including the cecum and the spleen.

Kaiser et al. theorized that these bacteria could be responsible for disease relapse. In support of this idea, they found that *S.* Tm. bacteria isolated from the cLN of ciprofloxacin-treated mice were still virulent and could establish infections when transferred into new mice. Further experiments showed that, following ciprofloxacin treatment, viable bacteria were found specifically inside “classical” dendritic cells, but not in another cLN cell type, the interstitial dendritic cells. As *S.* Tm. numbers in ciprofloxacin-treated cLN also changed in concert with the size of the classical dendritic cell population, the authors hypothesized that bacteria living inside dendritic cells form a reservoir that can reinstate infection upon withdrawal of the antibiotic. But how do these bacteria survive antibiotic treatment long enough to cause relapse?

Prior studies had shown that ciprofloxacin quickly kills *S.* Tm. cells in culture, and also that it easily accesses and penetrates body tissues. In fact, the authors' own control experiments confirmed that ciprofloxacin was able to reach the cLN and penetrate the cells within it, showing that the bacteria were not simply evading exposure to the antibiotic. This led Kaiser and colleagues to investigate the possibility that *S.* Tm. had instead become resistant to ciprofloxacin. Such resistance could arise from genetic changes that confer permanent partial or full resistance to the antibiotic, but *S.* Tm. cells isolated from ciprofloxacin-treated animals remained fully susceptible to ciprofloxacin when removed from the animal and grown in culture. Therefore, the researchers suspected that the bacteria's antibiotic resistance was not due to genetic changes, but was instead attributable to a phenotypic adaptation.

The literature indicated that many bacterial species can become tolerant to antibiotics by switching to slow rates of growth. To find out whether *S.* Tm. also acquires tolerance through slow growth rates, Kaiser et al. constructed a population dynamics–based simulation for cLN bacterial growth and compared its predictions to data from actual infections. This approach, and subsequent experiments tracking plasmid retention in *S.* Tm., demonstrated the existence of a slow-growing subpopulation of *S.* Tm. cells in the cLN. Ultimately, the authors concluded that such slow-growing cells, living within classical dendritic cells, are responsible for relapses in complicated *S.* Tm. infections.

There is irony in this situation because classical dendritic cells play a critical role in fighting bacterial infection. They seek out, phagocytose, and digest microbial invaders within peripheral tissues, then bring bacterial fragments to nearby lymph nodes for display to other immune cells. Yet *S.* Tm. somehow avoids digestion and—whether because of a change induced by phagocytosis or simply because of inherent variations within the bacterial population—a few of the bacteria slow their growth enough to resist the effects of antibiotic treatment. This led Kaiser and colleagues to investigate whether compounds that enhance immune activity could also improve bacterial clearance by ciprofloxacin. Indeed, they found that immune activators such as lipopolysaccharide and CpG dinucleotides reduced bacterial loads in the cLN of ciprofloxacin-treated mice. Altogether, these data explain how *S.* Tm. gains tolerance to ciprofloxacin, and point to possible improvements for treatment of complicated *S.* Tm. infections.

**Kaiser P, Regoes RR, Dolowschiak T, Wotzka SY, Lengefeld J, et al. (2014) Cecum Lymph Node Dendritic Cells Harbor Slow-Growing Bacteria Phenotypically Tolerant to Antibiotic Treatment.** doi:10.1371/journal.pbio.1001793

